# Urban particulate matter (PM) suppresses airway antibacterial defence

**DOI:** 10.1186/s12931-017-0700-0

**Published:** 2018-01-08

**Authors:** Xiaoyan Chen, Jinguo Liu, Jian Zhou, Jian Wang, Cuicui Chen, Yuanlin Song, Jue Pan

**Affiliations:** 1 Department of Pulmonary Medicine, Zhongshan Hospital, Fudan University and Shanghai Respiratory Research Institute, 180 Fenglin Road, Shanghai, 200032 People’s Republic of China; 2 Department of Infectious Diseases, Zhongshan Hospital, Fudan University, 180 Fenglin Road, Shanghai, 200032 People’s Republic of China

**Keywords:** Antimicrobial peptide, Bronchial epithelial cells, β-defensins, *Pseudomonas Aeruginosa*, Urban particulate matter

## Abstract

**Background:**

Epidemiological studies have shown that urban particulate matter (PM) increases the risk of respiratory infection. However, the underlying mechanisms are poorly understood. PM has been postulated to suppress the activation of airway epithelial innate defence in response to infection.

**Methods:**

The effects of PM on antibacterial defence were studied using an in vitro infection model. The levels of antimicrobial peptides were measured using RT-PCR and ELISA. In addition to performing colony-forming unit counts and flow cytometry, confocal microscopy was performed to directly observe bacterial invasion upon PM exposure.

**Results:**

We found that PM PM increased bacterial invasion by impairing the induction of β-defensin-2 (hBD-2), but not the other antimicrobial peptides (APMs) secreted by airway epithelium. PM further increases bacteria-induced ROS production, which is accompanied by an accelerated cell senescence and a decrease in bacteria-induced hBD-2 production, and the antioxidant NAC treatment attenuates these effects. The PM exposure further upregulated the expression of IL-8 but downregulated the expression of IL-13 upon infection.

**Conclusions:**

PM promotes bacterial invasion of airway epithelial cells by attenuating the induction of hBD-2 via an oxidative burst. These findings associate PM with an increased susceptibility to infection. These findings provide insight into the underlying mechanisms regarding the pathogenesis of particulate matter.

## Background

According to the World Health Organization (WHO), exposure to air pollution was a major global risk factor for diseases, accounting for approximately 7 million deaths in 2012 [1], particularly in developing countries where pneumonia was one of the most common diseases. Urban particulate matter (PM), which is a mixture of liquid and solid particles, is a major component of air pollution. PM consists of particles of varying sizes, including coarse (aerodynamic diameter ≤ 10 μm, PM10), fine (aerodynamic diameter ≤ 2.5 μm, PM2.5) and ultrafine (aerodynamic diameter ≤ 0.1 μm, PM0.1) particles [2].

Epidemiological studies have shown that PM exposure from vehicular emissions and cigarette smoke increased the vulnerability to respiratory infections [3–6]. Data from in vivo*/*in vitro experiments supported the deleterious effects of PM, such as its capacity to weaken the function of alveolar macrophages and airway epithelium cells, thereby increasing the risk of infection, such as pneumoniae [7, 8]. Furthermore, previous reports have shown that an increase in the PM2.5 exposure concentration was associated with an increased risk of *Pseudomonas aeruginosa* (*P. aeruginosa*) acquisition [9].

*P. aeruginosa* is one of the most common pathogens responsible for respiratory infections in hospitalized patients [10]. The phenomenon that PM exposure predisposes individuals to bacterial colonization and lung infections may suggest that PM weakens the respiratory host innate defence system, including the airway surface fluid (ASF) covering the airway epithelium. Classical components of the ASF that have antibacterial activity are lactoferrin, lysozyme, C-C motif chemokine ligand 20 (CCL-20), secretory leukocyte protease inhibitor (SLPI), LL-37/hCAP-18, and defensins. Human defensins (hBDs) have been identified as a critical part of the antimicrobial activity of the ASF [11].

hBDs, which are short amphiphilic cationic peptides, are abundant in the human airway and effective against bacteria, fungi and viruses [12, 13]. hBD-1-4 are vital players in airway epithelial innate defence. hBD-1 is constitutively expressed, whereas hBD-2 is inducible in response to stimulation [14]. hBD-2 mainly acts on gram-negative bacteria, such as *P. aeruginosa* [15]. hBD-2, but not hBD-1, has been reported to prevent and control infections not only by directly causing antimicrobial death but also by modulating the innate immune response [16]. Direct antimicrobial death has originally been attributed to lipid perturbations, which are a means of disrupting bacterial cell membranes and translocating bacteria to degrade internal targets [17]. Nevertheless, the immunostimulatory properties of the hBDs are highly diverse and play roles in cell survival, proliferation and migration, wound healing, angiogenesis and the induction of immune mediators, such as chemokines and cytokines [18, 19].

However, data regarding the effects of PM exposure on airway epithelial antibacterial defence are lacking. In this study, we mainly assessed the effects of PM on the expression of host airway antimicrobial peptides (AMPs) in response to *P. aeruginosa*. In addition, we investigated the possible mechanisms that altered the antibacterial defence molecules.

## Methods

### Reagents and cell culture

N-acetyl-l-cysteine (NAC, Sigma-Aldrich, St. Louis, MO, USA) and recombinant human β-defensin-2 protein (hBD-2, Abcam, Cambridge, UK) were used in this study. The PM (SRM NIST 1649b) was obtained from the Standard Reference Material Program and was certified by the National Institute of Standards and Technology (NIST, Gaithersburg, USA). Stock suspensions of PM (4 mg/ml) were prepared with PBS and further diluted in High Glucose Dulbecco’s Modified Eagle’s Medium (DMEM) to final concentrations of 10, 50, 100, 200 and 400 μg/ml. The human bronchial epithelial cell line (BEAS-2B), which was derived from normal human bronchial epithelial cells, was maintained at 37 °C in a humidified atmosphere containing 5% CO_2_ as published by Cachon et al. [20]

### *P. aeruginosa* experimental preparations

The *P. aeruginosa* PAO1 strain was chosen for this study due to its importance and prevalence in lung diseases, such as COPD and CF. PAO1 was grown in static Luria-Bertani Broth overnight. Then, the bacterial culture was diluted to 1:50 and placed on shaker for 3 h at 37 °C until the mid-log growth phase was reached. The harvested bacteria were washed thrice with PBS and diluted to the indicated concentrations.

### Bacterial invasion assay

We utilized the protocol described by Zaas et al. [21] with some modifications. First, BEAS-2B cells were seeded into 6-well plates at a density of 2.5 × 10^5^ cells/cm^2^. The cells were grown to approximately 50–60% confluency. Then, the cells were cocultured with PM for 24 h prior to the PAO1 infection at various multiplicities of infection (MOI) of 1, 10, or 20 by replacing the medium with DMEM containing the corresponding volumes of a bacterial suspension (OD600 0.25 = 1 × 10^8^ CFU/ml) for 2 h at 37 °C and 5% CO_2_. Then, the supernatants were removed, and the cells were washed thrice with PBS. In addition, 200 μg/ml gentamicin sulfate (Sigma-Aldrich, St. Louis, MO, USA) in 2 ml DMEM were added to the wells, followed by a 2-h incubation to eliminate the extracellular bacteria. Subsequently, the cells were washed five times and lysed with 2 ml of 0.1% Triton X-100 (Sigma-Aldrich, St. Louis, MO, USA). The lysates were serially diluted and plated onto Pseudomonas Cetrimide agar (OXOIDCM0579, Basingstoke, England) plates in triplicate. The numbers of internalized viable bacteria were counted according to the colony-forming units (CFU × 10^3^/ml).

### Invasion assay assessed by flow cytometry

BEAS-2B cells were pre-treated with NAC (5 mM for 1 h) and infected with green-fluorescent-protein (GFP)-labelled *P. aeruginosa* (GFP-PAO1, a gift from the University of California, San Francisco, USA) for 2 h at an MOI of 10. After 2 h of incubation, the cells were washed thrice with sterile PBS, and 200 μg/ml of gentamicin sulfate in 2 ml of DMEM were added to the wells, followed by a 2-h incubation to eliminate the membrane-bound bacteria. Subsequently, the intracellular cell fluorescence was measured by a FACScan flow cytometer (BD FACScantoTM, San Jose, CA, USA), which represented the number of intracellular bacteria [22, 23]. The flow cytometry data were analysed by FlowJo (vX.0.7, FlowJo, Ashland, OR, USA).

### Confocal microscopy

To directly evaluate the invasion of GFP-PAO1 by confocal microscopy, the invasion of *P. aeruginosa* was quantified as described previously with some modifications [24]. Briefly, the BEAS-2B cells were seeded on glass coverslips for 24 h. Then, the BEAS-2B cells on the coverslips were exposed to PM for 24 h with or without hBD-2 treatment according to the experimental design. Subsequently, GFP-PAO1 at an MOI of 10 was added to each well for 2 h. Following the infection, the cells were vigorously washed thrice with sterile PBS, and 200 μg/ml gentamicin sulfate were added to the wells, followed by a 2-h incubation. Then, the coverslips were fixed in 4% paraformaldehyde at room temperature for 15 min. To stain the nuclei, 4′, 6-diamidino-2-phenylindole (DAPI, Sigma-Aldrich, St. Louis, MO, USA) was incubated with the cells for 5 min. Subsequently, the cell membranes were stained with 1,19-dioctadecyl-3,3,39,39-tetramethylindocarbocyanine perchlorate (DiI, Beyotime, Jinan, China) at 37 °C for 10 min, and the coverslips were examined under a Leica TCS SP5 II confocal microscope (Wetzlar, Germany). Then, the GFP-PAO1 located in the intracellular BEAS-2B cells was counted. At least 100 cells were counted to quantify the average intracellular PAO1. The PAO1 invasion is expressed as the mean number of GFP-PAO1 per BEAS-2B cell (intracellular PAO1/cell).

### Cell viability assay

The cell viability was assayed by a Cell Counting Kit-8 (CCK-8) (Beyotime, Jinan, China). Briefly, the BEAS-2B cells were seeded in 96-well plates overnight and then treated with PM at doses of 0, 10, 50, 100, 200 and 400 μg/ml for 24 h. After the treatment, 10 μl of the CCK-8 solution were added to each well, and the plate was incubated for another 1 h at 37 °C. The absorbance was read by a microplate reader (FlexStation® 3; Molecular Devices, California, USA) at 450 nm. Each assay was repeated three times.

### Assessment of ROS formation and PM uptake

The intracellular reactive oxygen species (ROS) was determined using an oxidation-sensitive fluorescent probe (DCFH-DA). Briefly, BEAS-2B cells were plated in a 6-well plate at a density of 2.5 × 10^5^ cells per well and allowed to attach for 24 h. The culture medium was replaced with or without fresh culture media containing NAC (5 mM for 1 h) before adding various concentrations of PM. Then, the cells were infected with PAO1 after 24 h. After the PAO1 infection, the cells were washed with a PBS solution twice and incubated with DCFH-DA at a final concentration of 10 μmol/L for 20 min at 37 °C in the dark. Then, the fluorescence of 2,7-dichlorofluorescein (DCF) was determined using a FACScan flow cytometer (BD FACScanto™, San Jose, CA, USA). For each sample, 10,000 events were collected. The cellular uptake of PM was detected simultaneously and expressed as the side scatter (SSC) intensity [25].

### Analysis of gene expression using quantitative RT-PCR

The cells were harvested, and the total RNA was isolated using TRIzol^®^ reagent (Life Technology, Carlsbad, CA, USA) and quantified using a spectrophotometer (DeNovix DS-11, Wilmington, USA). Then, the RNA was reverse transcribed into cDNA using the ReverTra Ace^®^ qPCR RT Master Mix (TOYOBO, Osaka, Japan). PCR amplification was performed using a SYBR^®^ Green Realtime PCR Master Mix-Plus-Kit (TOYOBO, Osaka, Japan) on a 7500 Real Time PCR System (Applied Biosystem®, Life Technology). More information regarding the primers and probes is provided in Additional file 1.

### Enzyme-linked immunosorbent assay (ELISA)

To investigate the effects of PM exposure on the induction of hBD-2 by bacteria, ELISA assays were conducted to detect the hBD-2 levels in the supernatant. Briefly, BEAS-2B cells were seeded in 6-well culture plates at a density of 2.5 × 10^5^ cells/well in 2 ml of culture medium for 24 h. NAC was added to the supernatant 24 h prior to the 1-h PM exposure. Then, the cells were or were not infected with PAO1 according to the design of the experiment. ELISA kits (Kelei biological Technology, Shanghai, China) were used to measure the protein levels of hBD-2 in the cell culture supernatant according to the manufacturer’s protocol. The absorbance was read at 450 nm using a microplate reader (FlexStation^®^ 3; Molecular Devices, California, USA).

### Senescence-associated β-galactosidase assay (SA-β-gal)

Human BEAS-2B cells were seeded in 6-well culture plates for 24 h. Before detecting the activity of SA-β-gal using the senescence-associated β-galactosidase-staining-kit (Beyotime, Jinan, China) (according to the manufacturer’s instructions), the cells were exposed to PM at various concentrations for 24 h. Then, the cells were fixed with paraformaldehyde at room temperature and washed three times with PBS after 15 min. The cells were incubated with the SA-β-gal staining solution at 37 °C overnight. The level of SA-β-gal was determined by capturing the image using an Olympus IX51 (Olympus, UK).

### Assessment of the degree of senescence

All staining results were reviewed by two pathologists in a double-blind manner. At least 200 cells, including stained and unstained cells, were scored. The intensity and percentage of cells stained were scored under a × 400 field as previously described by C Huang et al. [26] The staining intensity was defined as 0 (no staining), 1 (weak staining), 2 (distinct staining), or 3 (very strong staining). A value called ‘HSCORE’ was calculated for each group using the following algorithm: HSCORE = *∑* (I × PC). In this algorithm, I and PC represent the intensity and percentage of stained cells at each intensity (0~100%), respectively. The scores range from 0 to 300.

### Statistical analysis

All results are expressed as the mean ± SD. The differences between the control and treated samples were compared by performing a t-test. *P* < 0.05 was considered statistically significant. All statistical analyses were performed using Prism software version 6 (GraphPad Software, San Diego, USA).

## Results

**Fig. 1:**
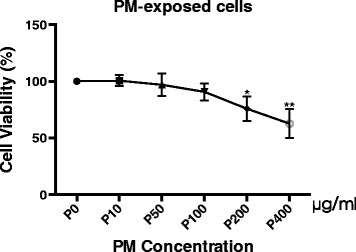
PM at a low concentration does not decrease cell viability. The cell viability of BEAS-2B cells after urban particulate matter (PM) exposure was assayed using a CCK-8 assay according to the manufacturer’s instructions. The cells were incubated for 24 h with various concentrations of PM as follows: 10, 50, 100, 200 and 400 μg/ml. The data are expressed as the mean ± SD of three independent experiments. ^*^P < 0.05 and ^**^P < 0.01 versus P0 (0 μg/ml)

Human bronchial epithelial cells were exposed to PM at concentrations of 0 (negative control), 10, 50, 100, 200 and 400 μg/ml for 24 h (Fig. 1), and their viabilities were assessed using a CCK-8 assay. The exposure of the BEAS-2B cells to PM at the low concentrations of 10, 50, and 100 for 24 h did not decrease the mitochondrial activity, which is a measure of the cell viability. These results were consistent with the reports published by Ekstrand-Hammarstrom et al. [27] However, following exposure to PM concentrations that surpassed 200 μg/ml, the cell viability was significantly impaired. These results indicate that PM at a low concentration does not decrease the cell viability.

**Fig. 2:**
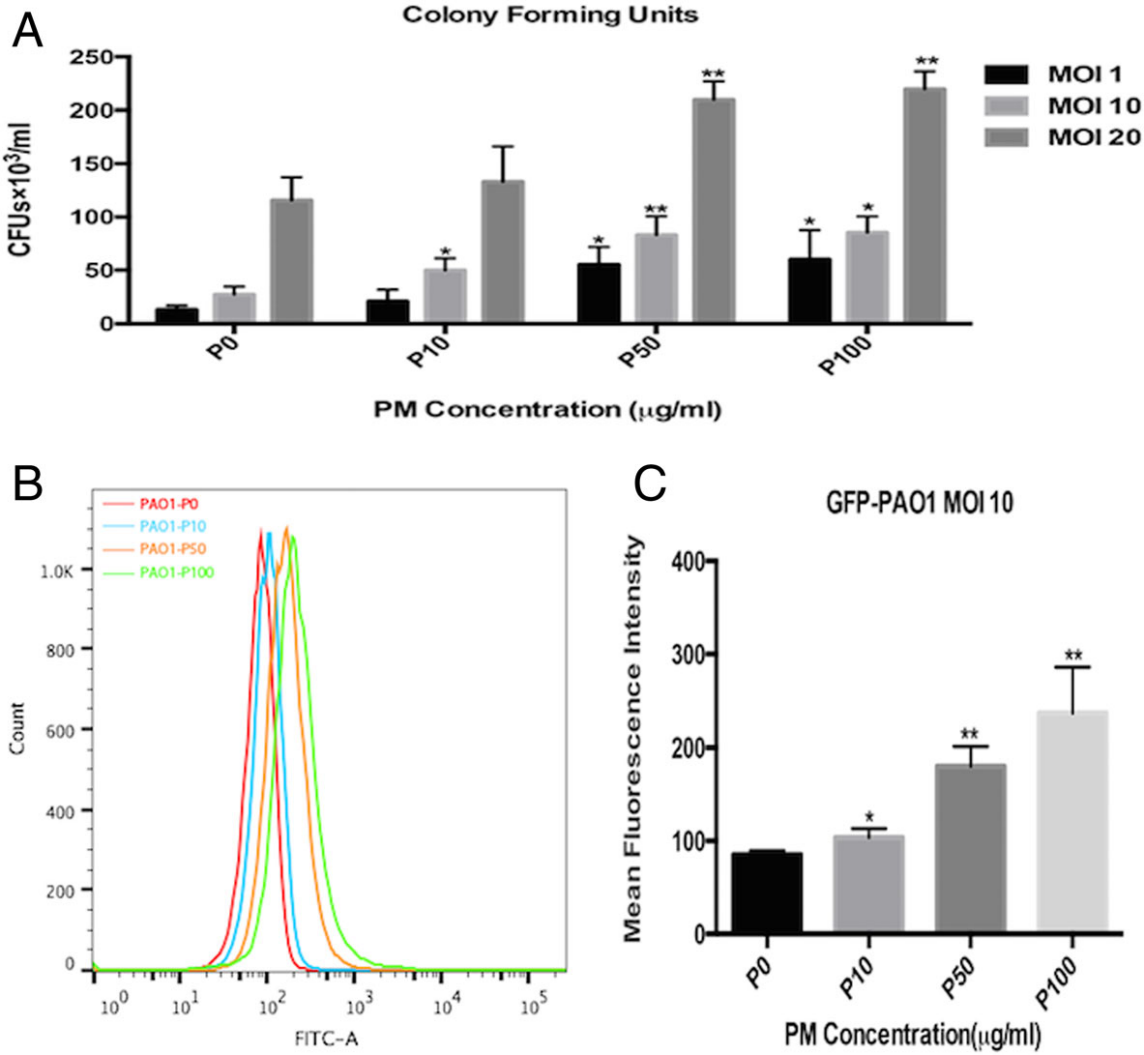
PM exposure impairs the antimicrobial defence in airway epithelial cells to promote *P. aeruginosa* invasion. **a** BEAS-2B cells were incubated with 0, 10, 50, or 100 μg/ml of PM for 24 h, followed by infection with *P. aeruginosa* at various MOI of 1, 10, or 20 for an additional 2 h. CFU counts were performed to determine the number of viable *P. aeruginosa*. **b** and **c** BEAS-2B cells were incubated with 0, 10, 50, or 100 μg/ml of PM for 24 h, followed by infection with GFP- *P. aeruginosa* at an MOI of 10 for an additional 2 h. The mean MFI represents the intracellular GFP-*P. aeruginosa*. The results are expressed as the mean ± SD of three independent experiments. CFUs, colony-forming units; MFI, mean fluorescence intensity; MOI, multiplicities of infection. *P < 0.05 and **P < 0.01 versus P0 (0 μg/ml)

The effects of PM on the response of human bronchial epithelial cells to *P. aeruginosa* were investigated. The cells were exposed to various concentrations of PM (10, 50, or 100 μg/ml) prior to *P. aeruginosa* infection (MOI of 1, 10, and 20), and the CFUs were counted. To ensure that the results of the bacterial invasion assay were not affected by extracellular *P. aeruginosa*, 200 μg/ml gentamicin sulfate were added to eliminate the extracellular bacteria, and multiple washing steps were performed. Thus, the CFUs assessed in our experiments represent intracellular *P. aeruginosa* and are not contaminated by extracellular bacteria remaining from the experimental infection process. The CFUs of *P. aeruginosa* were significantly higher in the PM-exposed groups (at an MOI of 10) than those in the unexposed (0 μg/ml) group in a concentration-dependent manner (Fig. 2a), which was also confirmed by flow cytometry (Fig. 2b and c). Furthermore, following exposure to 50 or 100 μg/ml PM prior to *P. aeruginosa* infection, an increase in the CFUs was observed, even at an MOI of 1. These data supported that PM exposure resulted in a significant increase in intracellular viable bacteria (Fig. 2a).

**Fig. 3:**
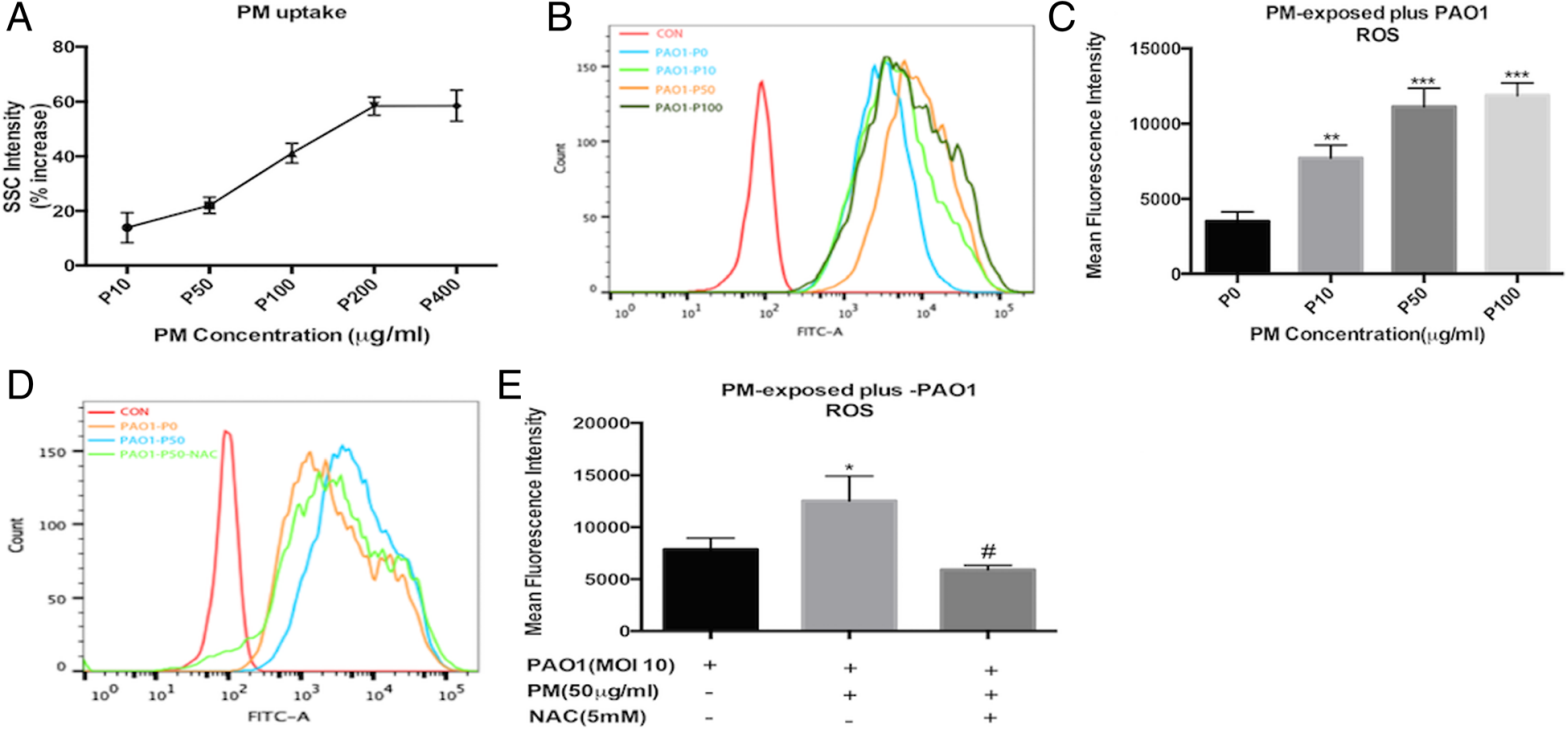
Effects of PM on intracellular ROS generation upon *P. aeruginosa* infection. **a** PM uptake was quantified according to the parameter of side scatter (SSC) intensity in BEAS-2B cells. **b** and **c** Intracellular ROS formation is expressed as the DCF mean fluorescence intensity (MFI), and the effect of NAC (**d** and **e**) was assessed in GFP-*P. aeruginosa*-infected BEAS-2B cells. PM significantly dose dependently increased the intracellular ROS generation upon PAO1-infection (**b**, **c**, **d** and **e**) in BEAS-2B cells. The results are expressed as the mean ± SD of three independent experiments. NAC, N-acetylcysteine; PM, PM-exposed cells; ROS, reactive oxygen species; ^*^P < 0.05 and ^**^P < 0.01 versus P0 (0 μg/ml)

Side scatter (SSC) intensity assessed by flow cytometry can provide information regarding the internal organelles and structure [25, 28]. A concentration-dependent increased uptake of PM by BEAS-2B cells was observed (Fig. 3a). This result was consistent with the observations using inverted microscopy. Furthermore, an investigation of oxidative stress in the PM-exposed plus *P. aeruginosa*-infected cells was conducted by detecting the intracellular ROS production. Our data show that PM dose-dependently upregulates *P. aeruginosa*-induced oxidative stress (Fig. 3b–e). Altogether, we conclude that as the PM uptake increases, the BEAS-2B cells produce more ROS upon *P. aeruginosa* infection.

**Fig. 4:**
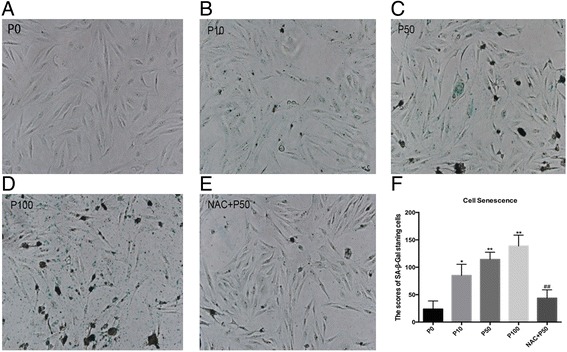
PM exposure induced cell senescence via oxidative stress damage. Senescence in BEAS-2B cells after PM exposure was detected by SA-β-gal staining. BEAS-2B cells cultured in a 6-well plate were incubated in medium containing various concentrations of PM as follows: 0, 10, 50, 100 μg/ml, and 50 μg/ml with 5 mM NAC. **a**, **b**, **c**, **d** and **e** Representative images (×200) of senescent cells assayed by SA-β-gal staining. **f** Quantitative analysis of senescent cells. The data are expressed as the mean ± SD of three independent experiments. NAC, N-acetylcysteine; PM, PM-exposed cells. ^*^P < 0.05, ^**^P < 0.01, and ^***^P < 0.001 versus P0 (0 μg/ml). ^#^*P* < 0.05 versus P50 (50 μg/ml)

Using the SA-β-gal staining assay, which is a well-established biomarker of cell senescence, we investigated PM-induced cell senescence in BEAS-2B cells and graded each group according to the staining intensity [26]. The score ranges from 0 to 300. As shown in Fig. 4a–d, the ultimate scores of the BEAS-2B cells exposed to 0, 10, 50, or 100 μg/ml for 24 h were 23.67 ± 8.570, 85.00 ± 11.85, 114.3 ± 7.5353, and 138.7 ± 11.57, respectively. Thus, we concluded that PM induced cell senescence in a concentration-dependent manner in the BEAS-2B cells. This observation is consistent with former reports [29]. Furthermore, the NAC pre-treatment decreased the proportion of SA-β-gal stained cells in the 50 μg/ml PM treatment group to 43.67 ± 8.762 (Fig. 4e). This result indicated that PM induced cell senescence, likely via oxidative stress damage.

**Fig. 5:**
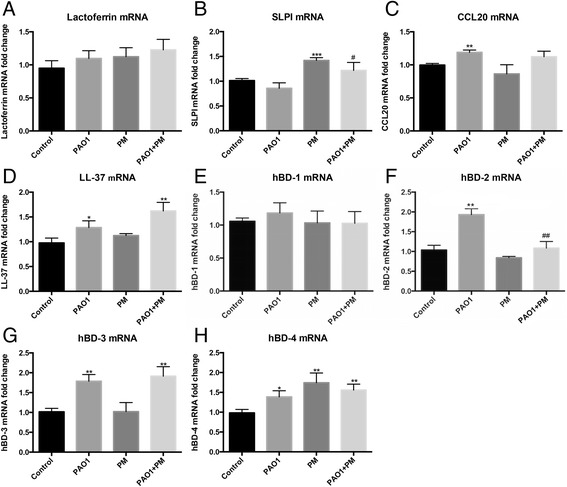
The mRNA expressions of the antimicrobial peptides in airway epithelial cells. The mRNA expressions of the antimicrobial peptides in airway epithelial cells were detected by RT-PCR. **a** Lactoferrin; **b** SLPI; **c** CCL- 20; **d** LL-37; **e** hBD-1; **f** hBD-2; **g** hBD-3; **h** hBD-4. Control, untreated cells; PAO1, infected cells; PM, PM-exposed cells; PAO1 + PM, exposed to exposed to PM and subsequently infected with P. aeruginosa; SLPI, secretory leukocyte peptidase inhibitor; CCL-20, C-C motif chemokine ligand 20; hBD-1-4, human defensin-1-4. The data are expressed as the mean ± SD of three independent experiments. *P < 0.05, **P < 0.01, and ***P < 0.001 versus Control. #*P* < 0.05 and ##*P* < 0.01 versus PM

**Fig. 6:**
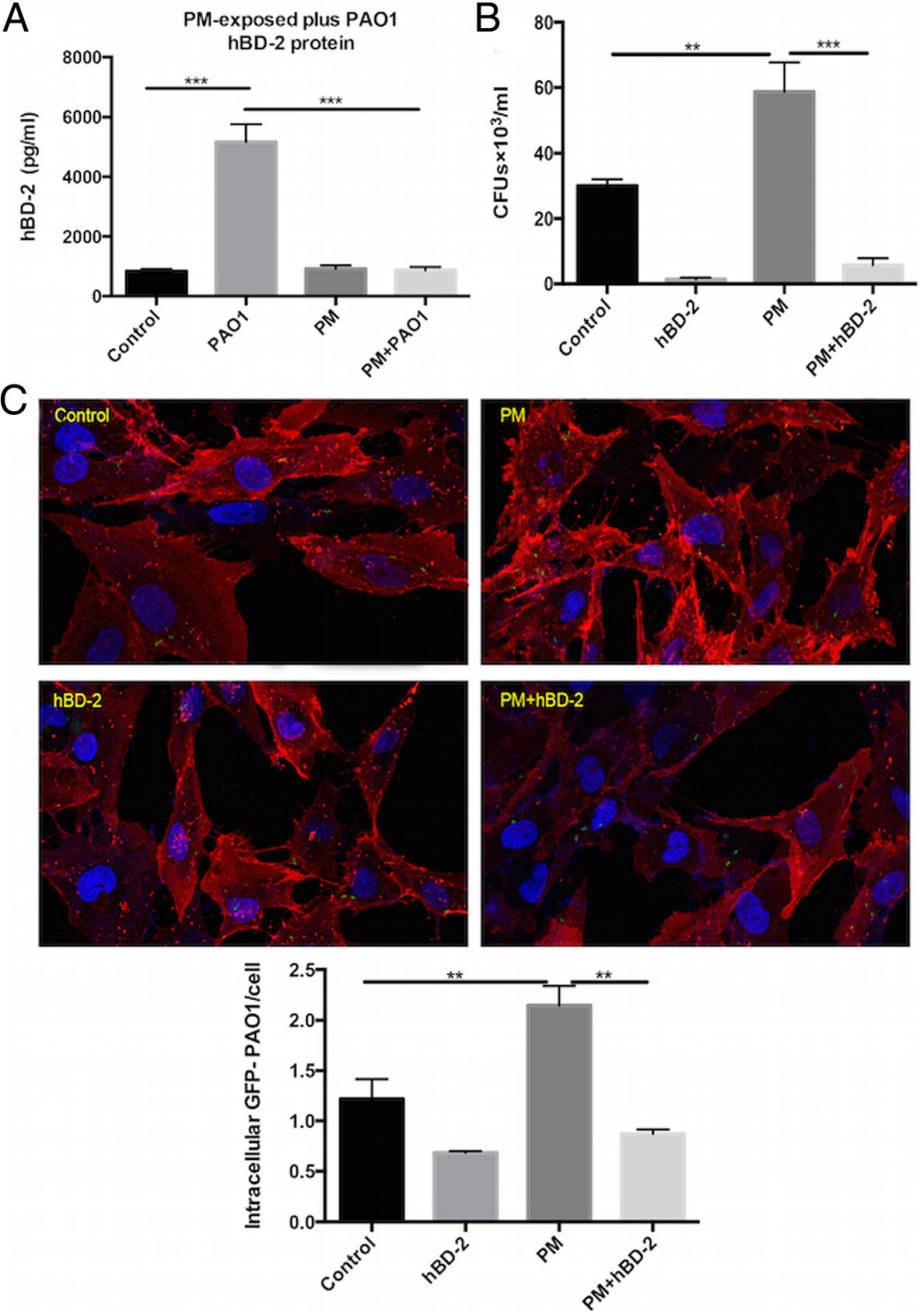
hBD-2 plays a critical role in PM epithelial host defence. **a** hBD-2 peptide concentration in the supernatant was assessed by ELISA. Control, untreated cells; PAO1, infected cells; PM, PM-exposed cells; PAO1 + PM, exposed to exposed to PM and subsequently infected with *P. aeruginosa*. **b** BEAS-2B cells were exposed to PM and subsequently infected with *P. aeruginosa*. External application of the hBD-2 peptide (5 μg per well) restored the epithelial antimicrobial activity by reducing the number of CFUs and intracellular bacteria (**c**). CFUs, colony-forming units. The results are expressed as the mean ± SD of three independent experiments. ^*^P < 0.05, ^**^P < 0.01, and ^***^P < 0.001

To investigate whether the defect of airway epithelial antibacterial defence upon PM exposure is related to a decreased induction of antimicrobial peptides, hBDs and other AMPs, which have been reported to express in the airway epithelium, were measured by RT-PCR. We found that the BEAS-2B cells did not express lysozyme, human defensin-5 (HD-5), and human defensin-6 (HD-6) (data not shown). Lactoferrin (Fig. 5a) and hBD-1 (Fig. 5e) were not induced by neither PM nor *P. aeruginosa*. Although SLPI (Fig. 5b) and hBD-4 (Fig. 5h) were upregulated by PM, they were not changed in PM-exposed plus *P. aeruginosa*-infected condition. In addition, the expression levels of CCL20 (Fig. 5c), LL-37/ hCAP-18 (Fig. 5d), hBD-2 (Fig. 5f), hBD-3 (Fig. 5g) and hBD-4 (Fig. 5h) were enhanced following *P. aeruginosa* treatment; however, no further changes were observed following the co-incubation with PM except for hBD-2. The suppressed induction of hBD-2 mRNA expression was consistent with the decline in hBD-2 peptide secretion (Fig. 6a). The external application of the recombinant hBD-2 peptide significantly reduced the number of CFUs (Fig. 6b) and intracellular bacteria (Fig. 6c) in the PM-exposed cells.

**Fig. 7:**
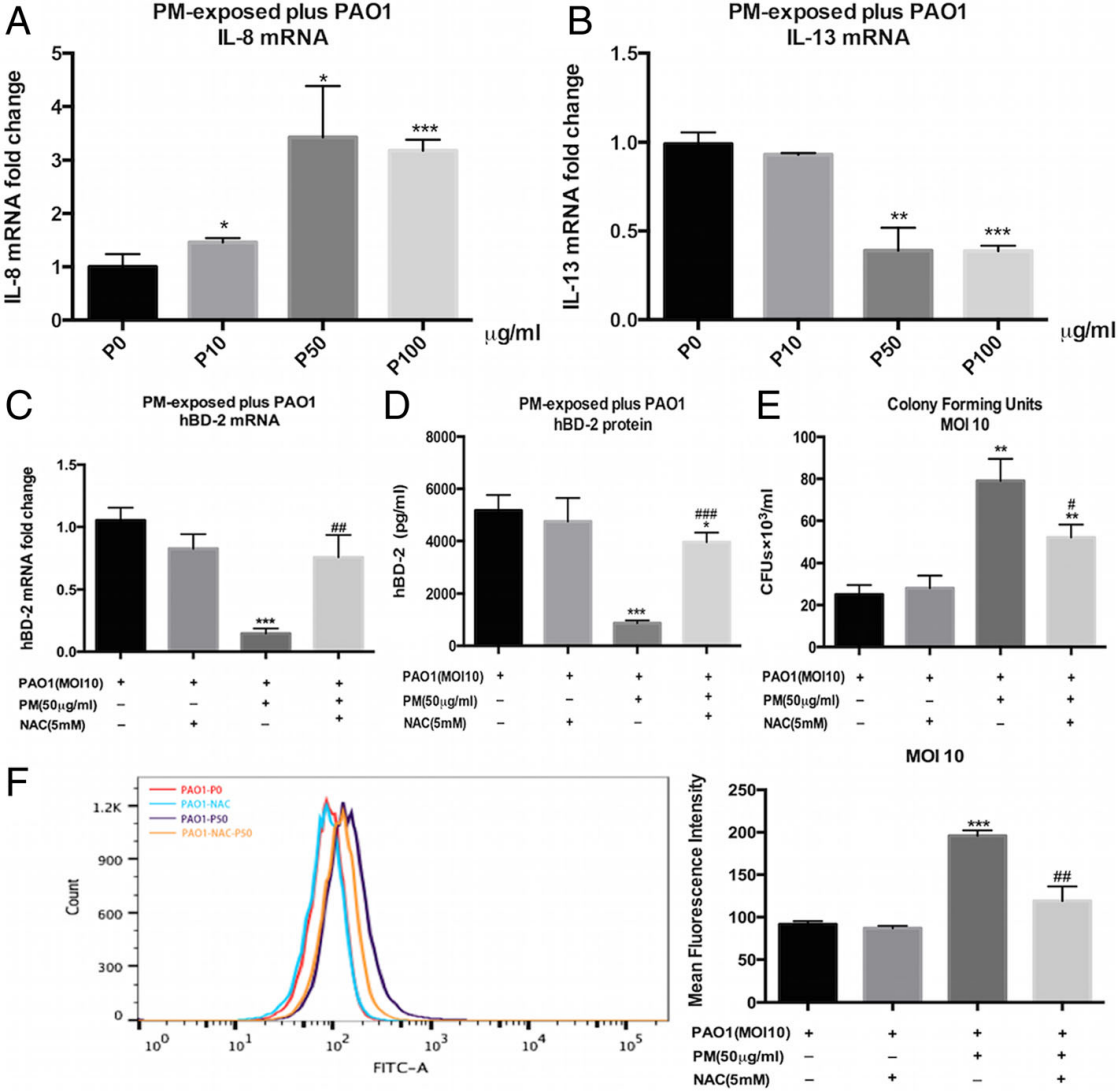
Effects of oxidative stress on the PM-dependent inhibition of airway epithelial antibacterial defence. **a** PM exposure further upregulated the expression of IL-8 but (**b**) downregulated the expression of IL-13. **c** and **d** The impeded induction of hBD-2 was partially reversed by NAC. Pre-treatment with NAC partially attenuated the PM-dependent increase in the CFU counts (**e**) and intracellular GFP-PAO1 (**f**). The results are expressed as the mean ± SD of three independent experiments. *P < 0.05, **P < 0.01, and ***P < 0.001 versus P0 (0 μg/ml). ^##^*P* < 0.01 versus P50 (50 μg/ml). NAC, N-acetylcysteine; IL, Interleukin; PM, PM-exposed cells; CFUs, colony-forming units

To determine whether the PM exposure led to a change in cell inflammation responses, we detected the effects of PM on the *P. aeruginosa*-induced IL-8 and IL-13 expression. PM dose dependently elevated the expression of the proinflammatory cytokine IL-8 in the *P. aeruginosa*-infected cells (Fig. 7a) and decreased the levels of the anti-inflammatory cytokine IL-13 (Fig. 7b). These findings are consistent with former reports [20, 27, 30, 31]. To determine whether ROS are involved in the mechanism of hBD-2 suppression, NAC, which is an antioxidant, was applied prior to the PM exposure. NAC not only partly abrogated the effects of PM on the induction of hBD-2 mRNA expression (Fig. 7c) and secretion (Fig. 7d) but also attenuated the bacterial invasion upon PM exposure (Fig. 7e and f). Thus, the downregulation of hBD-2 upon PM exposure promotes bacterial invasion through ROS-mediated senescence.

## Discussion

The main finding of this study is that PM disturbs the activation of the airway antibacterial defence. The PM-stimulated increased *P. aeruginosa* invasion is mainly due to the downregulation of hBD-2, but not a global impairment of AMPs. Oxidative stress is involved in this process because PM further increases bacteria-induced ROS production, which is accompanied by a decrease in bacteria-induced hBD-2 production, and the antioxidant NAC treatment attenuates these effects. In addition, both an imbalance of anti-inflammatory/ inflammatory mediator and a ROS-induced cell senescence contribute to the weakened antibacterial defence upon PM exposure. These results are consistent with reports by Sigaud who showed that exposure to ambient particles causes an inflamed alveolar milieu in which oxidative stress impairs antibacterial function in alveolar macrophages and diminishes bacterial clearance [22].

In China, the average concentrations of PM2.5 and PM10 are 94.1 ± 64.1 μg/m^3^ and 156.9 ± 99.2 μg/m^3^, respectively [32]. Considering the air volume (20 m^3^) inhaled daily by an individual, the 12.4 ml volume of the lung epithelial lining fluid, and the deposition rate (10%) of PM in the lung [33], we estimated that the average concentration of PM deposited in the lining fluid is approximately 40 μg/ml. If PM deposited in “hot spots” [33], the concentration could be much higher locally. In addition, the PM concentrations monitored in urban settings are higher than the concentrations used in our study, which indicated that the PM effects on the antibacterial immunity in our experiments were not due to exaggerated experimental conditions.

PM is known to impair the function of professional immune cells, such as macrophages [7, 22, 34], polymorphonuclear granulocytes (PMNs) [22], natural killer cells [23], and lymphocytes [35]. In this study, the innate defence function of the airway epithelium was also compromised by PM. PM exposure predisposes individuals to lung infections [3–6], suggesting that PM may weaken the respiratory antimicrobial defence, such as β-defensins.

Accumulating data support the vital role played by antimicrobial peptides in airway innate immunity due to their extensive antimicrobial activity [13]. An in vitro experiment [36] demonstrated that PM inhibited the LPS-induced increase in antimicrobial peptide, resulting in an impaired innate defence against pathogens. Our data suggest that it varies greatly in the expressions of AMPs in BEAS-2B cell under PM-exposed plus *P. aeruginosa*-infected condition. Lactoferrin and hBD-1 were constitutively expressed in BEAS-2B cell. Although SLPI and hBD-4 were induced by PM, they were not changed in PM-exposed plus *P. aeruginosa*-infected condition. And the expression levels of CCL-20, LL-37/hCAP-18, hBD-2-4 were enhanced following the treatment with *P. aeruginosa*; however, no further changes were observed in cells co-incubated with PM except for hBD-2, suggesting that hBD-2 is the critical peptide involved in PM-stimulated bacterial invasion. The intricate responses to PM in infection condition confirms that the regulation patterns and roles of various AMPs in airway epithelial immune are quite different [11, 37]. The contribution of hBD-2 was also suggested by a study that showed that hBD-2, but not hBD-1, prevented and controlled infections not only by direct antimicrobial death but also by innate immune system modulation [16].

The induction of oxidative stress and an anti-inflammatory/inflammatory mediator balance are crucial for the health effects generated by particles [38, 39] related to particulates. Interestingly, PM further elevated the *P. aeruginosa*-induced proinflammation cytokine IL-8 expression, which was accompanied by a reverse change in the anti-inflammatory cytokine IL-13. These findings are consistent with former reports [20, 27, 30, 31] demonstrating that PM exposure promotes inflammatory responses. A recent study [40] also found that the bacterial colonization level is associated with an increased inflammatory mediator in the lung in mice. Meanwhile, NAC significantly increased the expression and secretion of hBD-2. Thus, oxidative stress links anti-inflammatory and inflammatory responses to the regulation of hBD-2 production. The combination of a broken airway epithelial barrier caused by ROS and an increase in intracellular bacteria due to decreased hBD-2 expression could lead to a vicious cycle of inflammation, structural destruction and infection.

Considering that PM at low concentrations of 10, 50, and 100 μg/ml for 24 h did not attenuate the cell viability but accelerated the cell senescence induced by ROS, we propose that the effect of PM on hBD-2 production was not due to an overall decline in cell viability. Instead, in this study, cell senescence resulting from a PM-induced oxidative burst is a potential mechanism impairing the epithelial host defence. There are limitations to our study. It is not clear in this study that why there are disparate effects on various AMPs and an understanding of those mechanisms requires further study. Our use of PM in an aqueous suspension does not fully imitate the exposure of epithelia to PM via deposition at an aerosol-tissue interface in vivo. Our model cannot assess the effects of other cells in the human airway immune defence system. Further in vivo studies are needed to provide a thorough understanding of the adverse effects of PM not only in the lung epithelial cells but also in the context of inherent and adaptive immunity.

## Conclusions

PM impairs airway epithelial defence by impeding the induction of hBD-2 via an oxidative burst, potentially causing increased susceptibility to infection. These findings provide insight into the underlying mechanisms regarding the pathogenesis of particulate matter.

## Abbreviations

AMPs: Antimicrobial peptides
ASF: Airway surface fluid
CCL-20: C-C motif chemokine ligand 20
CF: Cystic fibrosis
CFUs: Colony-forming units
COPD: Chronic obstructive pulmonary disease
ELISA: Enzyme-linked immunosorbent assay
hBD-1: Human β-defensin-1
hBD-2: Human β-defensin-2
hBD-3: human β-defensin-3
hBD-4: Human β-defensin-4
hBDs: Human β-defensins
HD5: Human β-defensin-5
HD6: Human β-defensin-6
NAC: N-acetyl-l-cysteine
PM: Particulate matter
ROS: Reactive oxygen species
RT-PCR: Quantitative Real-time-PCR
SLPI: Secretory leukocyte peptidase inhibitor
